# Relationship between Icodextrin use and decreased level of small low-density lipoprotein cholesterol fractioned by high-performance gel permeation chromatography

**DOI:** 10.1186/1471-2369-14-234

**Published:** 2013-10-26

**Authors:** Eiichiro Kanda, Masumi Ai, Asami Iwamoto, Mitsuyo Okazaki, Yoshitaka Maeda, Sei Sasaki, Masayuki Yoshida

**Affiliations:** 1 Department of Nephrology, Tokyo Kyosai Hospital, Nakameguro 2-3-8, Meguroku, Tokyo 153-8934, Japan; 2 Life Science and Bioethics Research Center, Tokyo Medical and Dental University, Yushima 1-5-45, Bunkyoku, Tokyo 113-8519, Japan; 3 Department of Nephrology, Tokyo Medical and Dental University, Yushima 1-5-45, Bunkyoku, Tokyo 113-8519, Japan; 4 Department of Insured Medical Care Management, Tokyo Medical and Dental University, Yushima 1-5-45, Bunkyoku, Tokyo 113-8519, Japan; 5 Professor emeritus of Tokyo Medical and Dental University, Yushima 1-5-45, Bunkyoku, Tokyo 113-8519, Japan; 6 Skylight Biotech Inc, 100-4 Sunada Iijima-aza, Akita 011-0911, Japan; 7 Department of Nephrology, JA Toride Medical Center, Hongo 2-1-1, Toride, Ibaraki 302-0022, Japan

**Keywords:** Peritoneal dialysis, Icodextrin, Low density lipoprotein, Atherosclerosis, Statin, Chromatography

## Abstract

**Background:**

Because of the absorption of glucose in peritoneal dialysis (PD) solution, PD patients show an atherogenic lipid profile, which is predictive of poor survival in PD patients. Lipoprotein subclasses consist of a continuous spectrum of particles of different sizes and densities (fraction). In this study, we investigated the lipoprotein fractions in PD patients with controlled serum low-density lipoprotein (LDL) cholesterol level, and evaluated the effects of icodextrin on lipid metabolism.

**Methods:**

Forty-nine PD patients were enrolled in this cross-sectional study in Japan. The proportions of cholesterol levels to total cholesterol level (cholesterol proportion) in 20 lipoprotein fractions were measured using an improved method of high-performance gel permeation chromatography (HPGPC).

**Results:**

Twenty-six patients used icodextrin. Although no significant differences in cholesterol levels in LDL and high-density lipoprotein (HDL) were observed between the patients using icodextrin (icodextrin group) and control groups, HPGPC showed that the icodextrin group had significantly lower cholesterol proportions in the small LDL (t-test, *p*=0.053) and very small LDL (*p*=0.019), and significantly higher cholesterol proportions in the very large HDL and large HDL than the control group (*p*=0.037; *p*=0.066, respectively). Multivariate analysis adjusted for patient characteristics and statin use showed that icodextrin use was negatively associated with the cholesterol proportions in the small LDL (*p*=0.037) and very small LDL (*p*=0.026), and positively with those in the very large HDL (*p*=0.040), large HDL (*p*=0.047), and medium HDL (*p*=0.009).

**Conclusions:**

HPGPC showed the relationship between icodextrin use and the cholesterol proportions in lipoprotein fractions in PD patients. These results suggest that icodextrin may improve atherogenic lipid profiles in a manner different from statin.

## Background

Cardiovascular disease (CVD) is one of the leading causes of death in patients on peritoneal dialysis (PD) [1,2]. One of the risk factors of CVD is dyslipidemia. The kidney disease outcomes quality initiative (K/DOQI) reported that 78.6% of PD patients need treatment for dyslipidemia [3].

Lipoprotein subfractions consist of a continuous spectrum of particles of different sizes and densities. Small dense low-density lipoproteins (LDLs) and high-density lipoproteins (HDLs) are related to CVD events [4,5]. PD patients tend to show high levels of total cholesterol, LDL cholesterol (LDL-C), triglyceride (TG), and small dense LDL-C with a low level of HDL cholesterol (HDL-C) [6].

Icodextrin is a starch-derived, branched, water-soluble glucose polymer, a colloid osmotic agent, and derived from maltodextrin. It is used in the form of an aqueous solution for PD. The effect of icodextrin on cholesterol level has not been established yet. According to some reports, icodextrin does not change total cholesterol level [7,8]. On the other hand, there are reports that icodextrin increases HDL-C level and decreases LDL-C and TG levels [9,10], and there is a report that icodextrin decreases small dense LDL levels [11]. TG level is reduced by icodextrin [9,10,12]. However, the effect of icodextrin on lipoprotein subclasses has not been fully clarified yet.

The typical lipid profile of PD patients is atherogenic. However, there has been no clinical trial that determines whether all PD patients with dyslipidemia should receive lipid treatment for the prevention of CVD. It is important to study in the detail the lipoprotein subclasses in PD patients to prevent the occurrence of CVD in these patients. We previously established a method of high-performance gel permeation chromatography (HPGPC), which can separate lipoproteins into 20 fractions, and reported that small LDL-C and very small LDL-C levels positively correlate with visceral fat area [13,14]. Therefore, the aim of this study is to investigate the lipid profiles at subclass levels in PD patients and evaluate the relationship between icodextrin and lipid profiles, particularly cholesterol levels, by HPGPC.

## Methods

### Study design and study population

This is a cross-sectional study of PD patients (more than 20 years old) treated at Tokyo Kyosai Hospital, Tokyo, Japan, and JA Toride Medical Center, Ibaraki, Japan, which was approved by the local ethics committees of Tokyo Kyosai Hospital and JA Toride Medical Center. The investigation was carried out at a time from October 2011 to December 2011 at outpatient clinic. Written informed consent was obtained from each patient. Patients were eligible for inclusion in this study if they were able to stably continue continuous ambulatory PD (37 patients) or automated PD (12 patients) as outpatients. They were treated with PD solutions containing dextrose (Dianeal-NPD-2, -NPD-4 1.5 or 2.5, Baxter Japan, Tokyo; Mid Peric or Mid Peric L 135 or 250, Terumo, Tokyo) and with or without a PD solution containing icodextrin (Extraneal, Baxter Japan, Tokyo). All the patients in this study who selected Baxter’s PD system had used a PD solution containing icodextrin since the start of their PD. Because of Japanese national health insurance restrictions, they used only one bag of icodextrin, 1.5 to 2 L, for 8 to 12 hours per day. We adhered to the Japanese Society for Dialysis Therapy guidelines for PD [15]. Dyslipidemia was diagnosed on the basis of the criteria of the Japan Atherosclerosis Society [16]. A high LDL-C level was treated in accordance with the evidence-based practice guideline 2009 for the treatment of chronic kidney disease established by the Japanese Society of Nephrology and clinical guidelines for the evaluation and treatment of cardiovascular complications in hemodialysis patients established by the Japanese Society for Dialysis Therapy [17,18]. Serum LDL-C level was maintained at less than 120 mg/dl by administration of statin. Statins were administered to 16 patients (pravastatin, 4 patients; atorvastatin, 4; rosuvastatin, 6; pitavastatin, 2). None of the patients was administered ezetimibe or fibrates. We excluded patients who had malignant diseases, infectious diseases, or severe liver diseases.

### Data

Patient demographics including age, gender, and history of diabetes mellitus (DM) as well as comorbid conditions were obtained from the medical records of the patients at each hospital. Blood samples were obtained from every patient after a 12 hour overnight fasting with an overnight dwell of PD solution containing 2.5% dextrose. Serum samples were dispensed into three tubes, one for routine serum biochemistry at each hospital, one for measurement of apolipoprotein B at SRL Inc., Tokyo, Japan, and one for HPGPC at Skylight Biotech Inc., Akita, Japan. The blood samples for HPGPC were frozen at -80°C immediately after their collection until use. Routine serum biochemistry was carried out by standard methods at each hospital. Apolipoprotein B was measured using an immunoassay kit (Apolipoprotein B; Sekisui Medical Co., Tokyo, Japan). Lipoprotein fractions were analyzed by HPGPC as previously described [13,14]. In brief, 4 μL of whole serum samples were injected into columns and continuously monitored at 550 nm after an online enzymatic reaction. The HPGPC is used to simultaneously monitor and obtain simultaneously cholesterol and triglyceride levels in lipoprotein fractions in a single injection of samples. In this study, only cholesterol levels in lipoprotein fractions were analyzed by a mathematical procedure with modified Gaussian curve fitting to resolve overlapping peaks: chylomicron (CM) [Fraction 1 (F1) and F2], very-low-density lipoprotein (VLDL) (F3 - F7), LDL (F8 - F13), and HDL (F14 - F20) (Table 1). The proportion of cholesterol level in each lipoprotein fraction to total cholesterol level (cholesterol proportion) was calculated using the following formula: Cholesterol proportion = cholesterol level in each lipoprotein fraction (mg/dl)/total cholesterol level (mg/dl). Although the classification of LDLs based on HPGPC is not the same as that based on nondenaturing gradient gel electrophoretic analysis by Austin et al, medium, small, and very small LDLs (F9 - F13) are consistent with the classification of small dense LDLs by Austin et al. [13,14,19].

**Table 1:** Definition for lipoprotein fractions

**Class**	**Subclass**	**Fraction**
CM		F1
		F2
VLDL	Large VLDL	F3
		F4
		F5
	Medium VLDL	F6
	Small VLDL	F7
LDL	Large LDL	F8
	Medium LDL	F9
	Small LDL	F10
	Very small LDL	F11
		F12
		F13
HDL	Very large HDL	F14
		F15
	Large HDL	F16
	Medium HDL	F17
	Small HDL	F18
	Very small HDL	F19
		F20

*Abbreviations*: *CM* chylomicron, *VLDL* very-low-density lipoprotein, *LDL* low-density lipoprotein, *HDL* high-density lipoprotein.

### Statistical analyses

Results are presented as mean±standard deviation (SD). Intergroup comparisons were performed using a chi-square test for categorical variables and the t-test for continuous variables. Univariate linear regression analysis was carried out to determine the relationships between total cholesterol level and clinical and biochemical characteristics of patients. Multivariate linear regression analysis was used to determine whether icodextrin use is independently associated with the cholesterol proportion level in each lipoprotein group adjusted for body mass index (BMI), DM, statin use, and total cholesterol level, and a factor that was previously selected in the univariate linear regression analysis. These analyses were conducted using SAS, version 9.2 (SAS, Inc., North Carolina, US). Statistical significance was defined as *p*<0.05.

## Results

### Patient characteristics

Forty-nine patients on PD were included as samples for analysis. Twenty-six patients used icodextrin (icodextrin group), and twenty-three patients did not (control group). Patient demographics including biochemical data are shown in Table 2. The causes of end-stage kidney disease were as follows: diabetic nephropathy in 21 patients, chronic glomerulonephritis in 16, IgA nephropathy in 4, nephrosclerosis in 4, and unknown in 4.

**Table 2:** Clinical and biochemical characteristics of patients in this study

	**All**	**Control**	**Icodextrin**	***p***
N (%)	49	23 (46.9)	26 (53.1)	
Male (%)	38 (77.6)	19 (82.6)	19 (73.1)	0.51
Age	64.1±11.8	64.9±12.5	63.5±11.4	0.69
Height (cm)	160.9±9.9	159.9±10.1	161.8±9.8	0.50
Weight (kg)	60.6±11.9	57.2±11.2	63.6±11.9	0.06
BMI	23.3±3.7	22.3±3.6	24.2±3.5	0.07
DM (%)	21 (42.8)	7 (30.4)	14 (53.8)	0.15
Dyslipidemia (%)	34 (69.4)	19 (82.6)	15 (57.7)	0.06
Statin use (%)	16 (32.6)	8 (34.8)	8 (30.8)	0.77
Creatinine (mg/dl)	10.3±3.4	10.0±3.5	10.6±3.3	0.48
Albumin (g/dl)	3.3±0.5	3.4±0.5	3.2±0.4	0.20
CRP (mg/dl)	0.41±1.0	0.45±1.3	0.38±0.6	0.80
Glucose (mg/dl)	116.1±30.6	109.4±24.2	122.2±34.8	0.15
PD vintage (months)	39.9±28.5	42.1±30.5	37.9±27.1	0.61
D/P Cr	0.63±0.13	0.56±0.13	0.68±0.11	0.002

Values are expressed as mean ± standard deviation. The values are compared between the groups by the chi-square test or t-test.

*Abbreviations*: *control* control group, *icodextrin* icodextrin group, *BMI* body mass index, *DM* diabetes mellitus, *CRP* C-reactive protein, *PD* peritoneal dialysis, *D/P Cr* dialysate/plasma creatinine ratio.

The icodextrin and control groups were matached for gender, age, BMI, serum calcium level, serum albumin level, C-reactive protein (CRP) level, and PD vintage. The dialysate/plasma creatinine ratio (D/P Cr) of the icodextrin group was higher than that of the control group. Although no statistically significant difference was found, more patients with DM were included in the icodextrin group. Marginally more patients with dyslipidemia were in the control group than in the icodextrin group (*p*=0.06). No statistically significant difference in statin use was observed.

### Cholesterol and TG levels in lipoproteins

**Figure 1:**
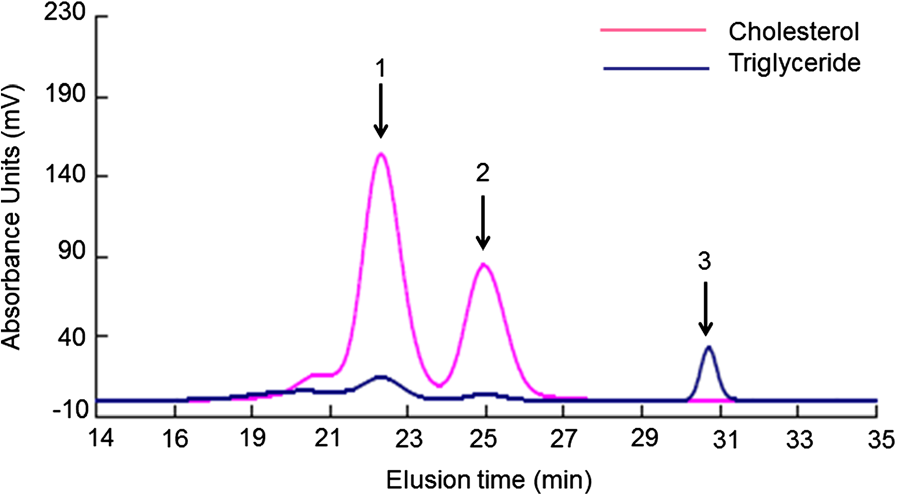
**Representative chromatographic pattern obtained by high-performance gel permeation chromatography.** The chromatography system could detect the cholesterol (red line) and triglyceride (blue line) levels in lipoprotein fractions from a diabetic male patient using icodextrin with a total cholesterol level of 175 mg/dl and a total triglyceride level of 53 mg/dl. In this study, the cholesterol levels in lipoprotein fractions were analyzed. Arrow 1 indicates the peaks for VLDL and LDL; arrow 2, HDL; arrow 3, endogenous free glycerol. Abbreviations: VLDL, very-low-density lipoprotein; LDL, low-density lipoprotein; HDL, high-density lipoproteins.

Serum samples from patients analyzed by HPGPC showed two separate peaks in the cholesterol profile and three peaks in the triglyceride profile. The first peak eluted at about 20–24 min contained VLDL and LDL and the second peak eluted at 24–27 min contained HDL (Figure 1). The third peak eluted at 30–31 min in the triglyceride profile showed endogenous free glycerol (Figure 1).

The differences in cholesterol level in lipoprotein classes and TG level were examined between the icodextrin and control groups (Table 3). No statistically significant differences in cholesterol and TG levels in lipoprotein classes were observed between the icodextrin and control groups (Table 3). The apolipoprotein B level in the icodextrin group was lower than that in the control group; this difference, however, did not reach statistical significance.

**Table 3:** Cholesterol levels in lipoproteins and TG levels

	**All**	**Control**	**Icodextrin**	***p***
Total cholesterol level (mg/dl)	183.1±37.2	184.8±43.9	181.6±31.0	0.77
CM cholesterol level (mg/dl)	1.1±1.8	1.2±2.4	0.9±1.2	0.55
VLDL-C level (mg/dl)	37.6±16.0	38.9±16.9	36.4±15.4	0.60
LDL-C level (mg/dl)	93.7±27.6	98.8±30.8	89.2±24.2	0.23
HDL-C level (mg/dl)	50.8±20.3	45.9±15.3	55.1±23.3	0.11
Triglyceride level (mg/dl)	139.3±70.6	145.2±81.5	134.1±60.6	0.59
Apolipoprotein B level (mg/dl)	91.6±24.3	97±26.5	86.8±21.5	0.15

Values are expressed as mean ± standard deviation. The values are compared between the groups by the chi-square test or t-test.

*Abbreviations*: *control* control group, *icodextrin* icodextrin group, *CM* chylomicron, *VLDL-C* very-low-density lipoprotein cholesterol, *LDL-C* low-density lipoprotein cholesterol, *HDL-C* high-density lipoprotein cholesterol.

**Figure 2:**
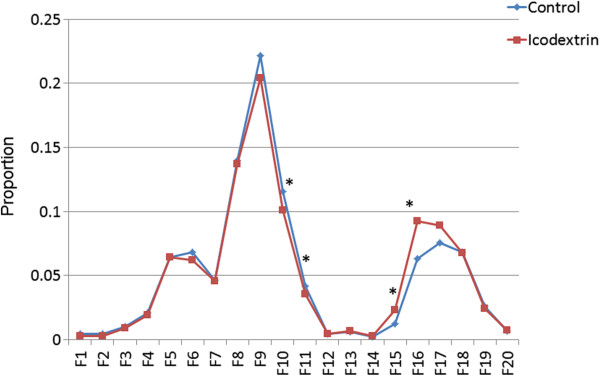
**Cholesterol proportions in each lipoprotein fractions in icodextrin group compared with those in control group.** The average cholesterol proportion in each lipoprotein fraction is indicated in the graph. The values are compared between the groups by the t-test. *, *p*<0.05. Abbreviations: proportion, average of the proportion of cholesterol level in each lipoprotein fraction to total cholesterol level; control, control group; icodextrin, icodextrin group; F1-20, Fraction 1-20.

The cholesterol proportion in each lipoprotein fraction is shown in Figure 2 and Table 4. In small LDL (F10) and very small LDL (F11), the cholesterol proportions in the icodextrin group were lower than those in the control group (F10, *p*=0.053; F11, *p*=0.019). In very large HDL (F15), and large HDL (F16), the cholesterol proportions in the icodextrin group were higher than those in the control group (F15, *p*=0.037; F16, *p*=0.066).

**Table 4:** Cholesterol proportions in lipoprotein fractions in icodextrin group compared with those in control group

**Class**	**Subclass**	**Fraction**	**ALL**	**Control**	**Icodextrin**	***p***
CM		F1	0.0036±0.0075	0.0045±0.01	0.0029±0.0036	0.47
		F2	0.0025±0.0043	0.0028±0.0055	0.0022±0.0029	0.63
VLDL	Large VLDL	F3	0.0095±0.01	0.01±0.012	0.0091±0.0087	0.78
		F4	0.02±0.019	0.021±0.021	0.019±0.018	0.73
		F5	0.064±0.026	0.064±0.028	0.064±0.024	0.96
	Medium VLDL	F6	0.065±0.023	0.068±0.025	0.062±0.021	0.35
	Small VLDL	F7	0.046±0.012	0.046±0.01	0.045±0.014	0.85
LDL	Large LDL	F8	0.14±0.033	0.14±0.036	0.14±0.031	0.77
	Medium LDL	F9	0.21±0.041	0.22±0.041	0.2±0.041	0.15
	Small LDL	F10	0.11±0.027	0.12±0.02	0.1±0.03	0.053
	Very small LDL	F11	0.038±0.0091	0.042±0.0069	0.035±0.0099	0.019*
		F12	0.0045±0.0034	0.0045±0.0033	0.0044±0.0035	0.93
		F13	0.0064±0.0011	0.0063±0.00092	0.0064±0.0013	0.82
HDL	Very large HDL	F14	0.0025±0.0015	0.0021±0.00099	0.0028±0.0018	0.11
		F15	0.018±0.018	0.013±0.009	0.023±0.022	0.037*
	Large HDL	F16	0.079±0.057	0.063±0.046	0.093±0.062	0.066
	Medium HDL	F17	0.083±0.028	0.076±0.026	0.089±0.029	0.1
	Small HDL	F18	0.068±0.017	0.068±0.016	0.068±0.018	0.94
	Very small HDL	F19	0.025±0.0059	0.026±0.0059	0.024±0.0059	0.36
		F20	0.007±0.0014	0.0069±0.0013	0.0071±0.0015	0.6

Values are expressed as mean ± standard deviation. The values are compared between the groups by the t-test. *, *p*<0.05.

*Abbreviations*: *icodextrin* icodextrin use, *statin* stain use, *CM* chylomicron, *VLDL* very-low-density lipoprotein, *LDL* low-density lipoprotein, *HDL* high-density lipoprotein, *cholesterol proportion* proportion of cholesterol level in each lipoprotein fraction to total cholesterol level.

### Independent effect of icodextrin on cholesterol proportions in lipoproteins

A single regression analysis showed that gender was associated with total cholesterol level (*p*=0.0035). Age; BMI; serum creatinine, albumin, and plasma glucose levels; PD vintage; and D/P Cr were not associated with total cholesterol level. Multivariate regression analysis adjusted for gender, BMI, total cholesterol level, DM, and statin use showed that icodextrin use was independently associated with cholesterol proportions (Table 5). In small LDL (F10) and very small LDL (F11), the cholesterol proportions were negatively associated with icodextrin use. In very large HDL (F15), large HDL (F16) and medium HDL (F17), the cholesterol proportions were positively associated with icodextrin use.

**Table 5:** Icodextrin use correlated with cholesterol proportions in lipoprotein fractions

			**Icodextrin**	
**Class**	**Subclass**	**Fraction**	**β**	***p***
CM		F1	-0.0026 (0.0027)	0.35
		F2	-0.0011 (0.0015)	0.47
VLDL	Large VLDL	F3	-0.0033 (0.0030)	0.27
		F4	-0.0063 (0.0055)	0.26
		F5	-0.0077 (0.0075)	0.31
	Medium VLDL	F6	-0.0094 (0.0071)	0.19
	Small VLDL	F7	-0.0023 (0.0039)	0.55
LDL	Large LDL	F8	-0.00057 (0.0096)	0.95
	Medium LDL	F9	-0.014 (0.012)	0.25
	Small LDL	F10	-0.017 (0.0079)	0.037*
	Very small LDL	F11	-0.0061 (0.0027)	0.026*
		F12	-0.00083 (0.00089)	0.36
		F13	0.00011 (0.00035)	0.76
HDL	Very large HDL	F14	0.00075 (0.00043)	0.087
		F15	0.012 (0.0055)	0.040*
	Large HDL	F16	0.036 (0.017)	0.047*
	Medium HDL	F17	0.020 (0.0075)	0.009*
	Small HDL	F18	0.0033 (0.0044)	0.45
	Very small HDL	F19	-0.00016 (0.0015)	0.92
		F20	0.00047 (0.00037)	0.20

Values are expressed as estimated parameter (standard error) and *p* values. The variables in multivariate linear regression analysis include gender, body mass index, DM, total cholesterol level, statin use, and icodextrin use. *, *p*<0.05

*Abbreviations*: *icodextrin* icodextrin use, *β* estimated parameter; *CM* chylomicron, *VLDL* very-low-density lipoprotein, *LDL* low-density lipoprotein, *HDL* high-density lipoprotein, cholesterol proportion, proportion of cholesterol level in each lipoprotein fraction to total cholesterol level; *DM* diabetes mellitus.

## Discussion

In this study, we were able to evaluate in detail the lipoprotein profile among PD patients with controlled serum LDL levels. Although no significant differences were observed in patient characteristics and cholesterol levels in LDL and HDL between the icodextrin and control groups, the icodextrin group showed significantly lower cholesterol proportions in small and very small LDLs, and significantly higher cholesterol proportions in very large and large HDLs. This study was partially consistent with previous reports that there are no significant differences in total cholesterol and TG levels between the icodextrin and control groups [7,8]. However, there has been no report on the details of the effects of icodextrin on lipid profiles. By HPGPC, we were able to determine the differences in lipid profiles between the icodextrin and control groups. These findings suggest a possibility that icodextrin may affect the lipid profiles at the fraction level in PD patients with controlled serum LDL levels.

In this study, we showed the relationship between icodextrin use and decreased cholesterol proportions in small LDL (F10) and very small LDL (F11). Our results suggest that icodextrin may be effective for reducing cholesterol levels in small and very small LDLs via a mechanism different from that for statins. TG level exerts a strong influence on LDL size [20]. Icodextrin has been reported to decrease TG level with decreased glucose absorption level in PD patients [9]. These reports suggest that icodextrin may decrease small LDL and very small LDL levels by decreasing TG level. Moreover, it has been reported that insulin resistance is associated with excess hepatic production of large VLDLs [21]. Large VLDLs are precursors of small dense LDLs [22]. Using icodextrin lowers insulin level and improves insulin resistance [23,24]. Therefore, it is suggested that there is another mechanism by which icodextrin decreases small LDL and very small LDL levels, that is, by improving insulin resistance.

Dyslipidemia is a risk factor for CVD in CKD patients. Statins are considered effective for treating dyslipidemia in dialysis patients [25]. However, clinical interventional studies of dialysis patients did not show the efficacy of statins in reducing the number of cardiovascular events [26-28]. A meta-analysis showed that statins had little effect on all-cause mortality, cardiovascular mortality, or cardiovascular events in dialysis patients [29]. Although the above research results suggest that lipid-lowering therapies are not effective in preventing the development of CVD in dialysis patients, their benefit should not completely be denied. There are various risk factors that contribute to CVD in PD patients, not only traditional risk factors, but also dyslipidemia, inflammation, homocysteine, and vascular calcification [30-32]. Because these factors are associated with each other, it is not sufficient to lower lipid levels by statins, and composite therapies are needed to prevent the progression of CVD in PD patients. It has been reported that icodextrin improves PD patient survival [33,34]. This study suggests that icodextrin improves the lipid profile in PD patients in a manner different from statins. From these results, icodextrin is a candidate therapy for the composite anti-CVD therapies after statins.

In this study, although conventional measurement of cholesterol levels in lipoprotein classes could not reveal the differences in lipid profiles between the icodextrin and control groups, measurement of cholesterol levels in lipoprotein fractions could detect a difference. There has been increasing evidence of the importance of more detailed lipid measurements. An observational study of hemodialysis patients showed that total cholesterol, LDL-C, and HDL-C levels are not associated with mortality, but that the smaller size of LDL particles is related to the poor prognosis of patients [35]. These results suggest a hypothesis that cholesterol levels in lipoprotein fractions predict more precisely a patient’s prognosis than those in lipoprotein classes. However, the role of detailed lipoprotein fractions in atherosclerosis is unclear in PD patients. This is a rationale to study the lipoprotein fractions in PD patients and whether lipid-lowering therapies in lipoprotein fractions improve the survival of PD patients with interventional icodextrin treatment.

This study has several limitations. First, as with any cross-sectional study, we were unable to examine the longitudinal changes in laboratory findings over time. Second, this study had only forty-nine patients. The statistical power of this study may not be sufficient for detecting the relationship between icodextrin use and lipoprotein fractions. Third, geographical and selection bias may have been included in this study. Fourth, we did not investigate the calorie intake such as diet and glucose load of PD solution. We were unable to adjust for the effects of calorie intake on dyslipidemia. Fifth, the lipid profiles are affected time-dependently by using the PD solution with icodextrin. A longitudinal study is needed to reveal the time-dependent effect of icodextrin on lipid profile. In this study, PD vintage was equal to the period of use of a PD solution with or without icodextrin. Sixth, there is a possibility that the absorbed icodextrin in samples may have caused measurement errors in serum glucose levels. However, because blood samples were obtained from every patient with an overnight dwell of PD solution containing 2.5% dextrose, and the glucose levels were not used in the regression models, the effect of icodextrin on the errors in serum glucose levels was decreased. Seventh, because most of the patients showed anuria, we were unable to precisely evaluate their urine volume.

## Conclusions

Among PD patients, the relationships between icodextrin use and cholesterol proportions in small and very small LDLs and HDLs were observed by HPGPC. These relationships were independent of statin use. Icodextrin may improve atherogenic lipid profiles in fraction level in a manner different from statin in PD patients.

## Abbreviations

PD: Peritoneal dialysis; LDL: Low-density lipoprotein; DM: Diabetes mellitus; HPGPC: High-performance gel permeation chromatography; HDL: High-density lipoproteins; CVD: Cardiovascular disease; K/DOQI: Kidney disease outcomes quality initiative; TG: Triglyceride; CM: Chylomicron; F1: Fraction 1; VLDL: Very-low-density lipoprotein; cholesterol proportion: proportion of cholesterol level in each lipoprotein fraction to total cholesterol level; SD: Standard deviation; BMI: Body mass index; icodextrin group: patients using icodextrin; control group: patients not using icodextrin; CRP: C-reactive protein; D/P Cr: Dialysate/plasma creatinine ratio.

## Competing interests

No financial or other interests to be declared.

## Authors’ contributions

Each author contributed to this manuscript. EK and AI collected the data. EK wrote the manuscript. EK, MA and MO contributed to the statistical analysis and interpretation of the data. YM, SS and MY contributed to the conception and design of the study and revised this study. All authors reviewed and approved the manuscript.

## Pre-publication history

The pre-publication history for this paper can be accessed here:

http://www.biomedcentral.com/1471-2369/14/234/prepub
